# The Role of Tenascin-C in Tissue Injury and Repair After Stroke

**DOI:** 10.3389/fimmu.2020.607587

**Published:** 2021-01-21

**Authors:** Takeshi Okada, Hidenori Suzuki

**Affiliations:** ^1^Department of Neurosurgery, Mie University Graduate School of Medicine, Tsu, Japan; ^2^Department of Neurosurgery, Kuwana City Medical Center, Kuwana, Japan

**Keywords:** biomarker, blood-brain barrier disruption, cerebral vasospasm, matricellular protein, neuroinflammation, neuronal apoptosis, stroke, subarachnoid hemorrhage

## Abstract

Stroke is still one of the most common causes for mortality and morbidity worldwide. Following acute stroke onset, biochemical and cellular changes induce further brain injury such as neuroinflammation, cell death, and blood-brain barrier disruption. Matricellular proteins are non-structural proteins induced by many stimuli and tissue damage including stroke induction, while its levels are generally low in a normal physiological condition in adult tissues. Currently, a matricellular protein tenascin-C (TNC) is considered to be an important inducer to promote neuroinflammatory cascades and the resultant pathology in stroke. TNC is upregulated in cerebral arteries and brain tissues including astrocytes, neurons, and brain capillary endothelial cells following subarachnoid hemorrhage (SAH). TNC may be involved in blood-brain barrier disruption, neuronal apoptosis, and cerebral vasospasm *via* the activation of mitogen-activated protein kinases and nuclear factor-kappa B following SAH. In addition, post-SAH TNC levels in cerebrospinal fluid predicted the development of delayed cerebral ischemia and angiographic vasospasm in clinical settings. On the other hand, TNC is reported to promote fibrosis and exert repair effects for an experimental aneurysm *via* macrophages-induced migration and proliferation of smooth muscle cells. The authors review TNC-induced inflammatory signal cascades and the relationships with other matricellular proteins in stroke-related pathology.

## Introduction

Stroke is a large public concern in terms of both human and financial resources (1, 2). In the United States, annual stroke expenses have reached approximately 33.9 billion dollars (2). Although recent research has been clarifying pathological changes in the brain following stroke, therapeutic options for these patients remain limited.

Neuroinflammation is a key pathologic change arising from stroke. Findings from both clinical and animal studies have indicated that inflammatory reactions may contribute to the development of brain injury following stroke (3–5). Post-stroke tissue damage releases secondary breakdown products of brain tissue and blood components. Damage-associated molecular patterns (DAMPs) are endogenous molecules released as a result of tissue damage that rapidly activate the innate immune response by interacting with a number of pattern recognition receptors (PRRs) located primarily on microglia and macrophages (6, 7). Activated microglia and macrophages release inflammatory cytokines and mediators *via* activation of signaling pathways downstream of the PRRs. The PRRs include Toll-like receptors (TLRs), cytosolic NOD-like receptors and inflammasomes, receptors for advanced glycation end products, and other scavenger receptors (8–10). Following stroke, the TLR4 signaling pathway is involved in the initial steps of neuroinflammation cascades, which result in brain injury such as vasogenic and cytotoxic edema and blood-brain barrier (BBB) disruption (11). Furthermore, neuroinflammation recruits more DAMPs, accelerating the inflammatory response. The secondary brain injury includes early brain injury (EBI), cerebral vasospasm (CVS), and delayed cerebral ischemia (DCI) after subarachnoid hemorrhage (SAH). Neuroinflammation is currently considered to be a critical factor contributing to morbidity and mortality in stroke patients who survive the initial brain damage and needs to be addressed in order to improve clinical outcomes (11–13).

Matricellular proteins (MCPs) are extracellular matrix (ECM) components upregulated and released by tissue damage, exerting both beneficial and harmful effects through binding to receptors, other matrix proteins, growth factors (GFs), and cytokines (14). Recent studies have demonstrated the efficacy of treatments targeting MCPs in preclinical stroke neuroinflammation models (15, 16).

In this review, we focus on a MCP tenascin-C (TNC) involved in neuroinflammation following stroke, and highlight current evidence for its use as a clinical biomarker and a therapeutic target.

## What Are MCPs?

The concept of MCPs was introduced in 1995 due to their characteristics which differ from classical ECM proteins (17). MCPs are currently considered important inducers that regulate the expression of inflammatory mediators and are involved in diverse pathological changes such as cell death, immunomodulation, inflammation, fibrosis, vascular permeability, and angiogenesis *via* modulation of the molecular functions or cellular responses to the molecules (18, 19). MCPs can work on the plasma membrane, intracellularly, in body fluids, or in the ECM, and also act as reservoirs of the bioactive molecules (18, 19). The level of protein expression is low in normal physiological conditions in adult tissues in general, and MCP knockout mice undergo normal development (18, 20). Almost all tissues and cell types produce MCPs following various stimuli which disappear after stimulus removal. MCPs do not provide a scaffold for stable cell adhesion, but induce cell motility and tissue remodeling *via* modulation of cell surface receptors, other matrix proteins, GFs, and cytokines (**Table 1**) (18). Accumulating evidence suggests that many types of MCPs, such as TNC, periostin, galectin-3, and osteopontin, contribute to aggravation or improvement of neuroinflammation in stroke at least partly by influencing the expression of each other (15, 18–23).

**Table 1:** Characteristics of matricellular proteins compared with classical extracellular matrix (ECM) proteins.

Classical ECM protein	Matricellular protein
- Structural protein	- Soluble non-structural protein - Low expression level under normal physiological conditions in adult tissues - Induced in almost any tissue by stimuli and disappear after stimulus removal
- Provide a scaffold for stable cell adhesion	- Induce cell motility and tissue remodeling - Various functions *via* interacting with cell surface receptors, other matrix proteins, growth factors, and cytokines - Knockout mice basically undergo normal development

## TNC: The Structure and Isoforms

**Figure 1:**
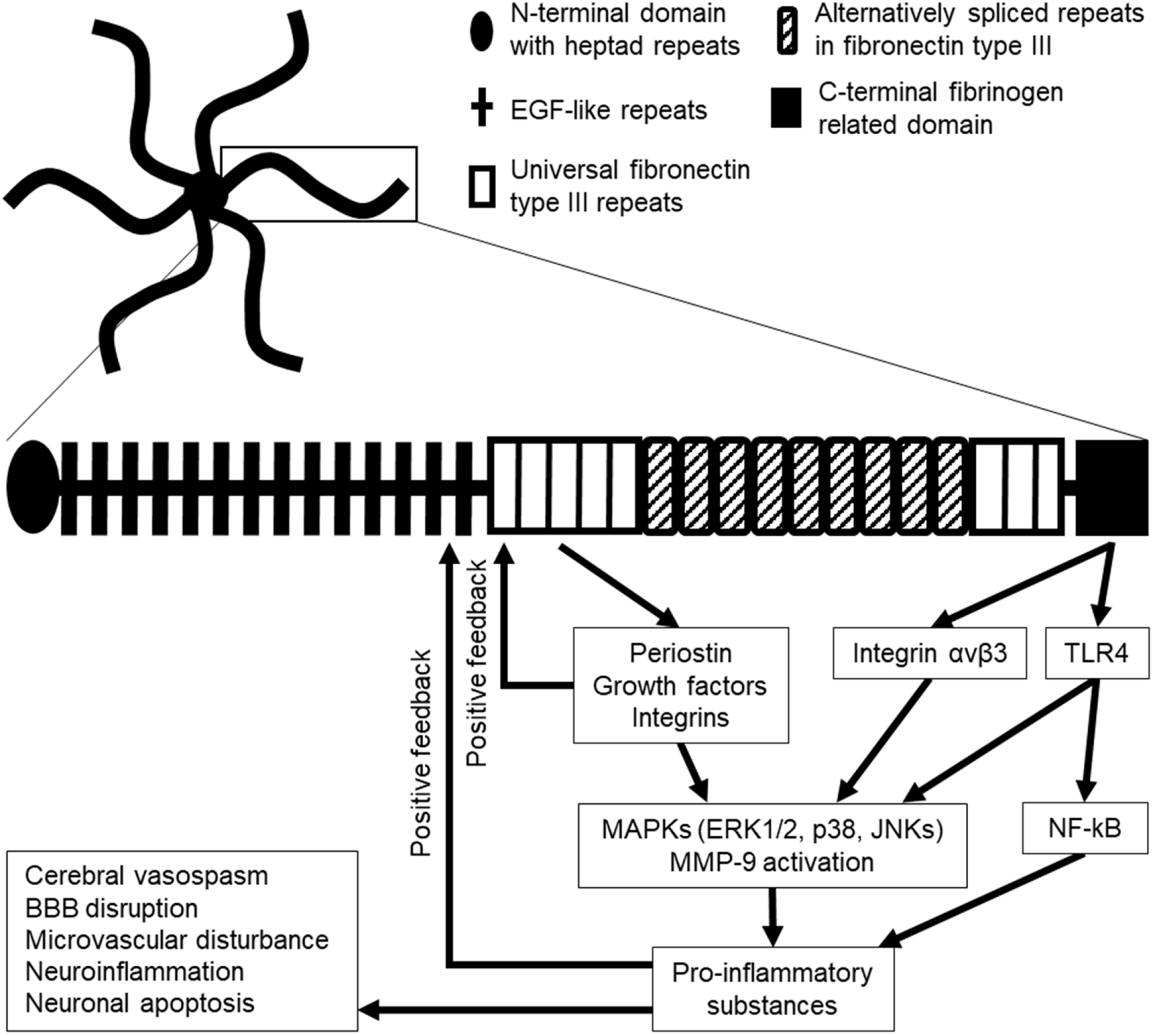
Hexamer structure of tenascin-C (TNC; upper), monomer structure of TNC (lower), and the possible downstream signaling pathway in stroke. Six TNC monomers combine to a hexamer at their N-terminal domains. EGF, epidermal growth factor; ERK1/2, extracellular signal-regulated kinase 1/2; JNKs, c-Jun N-terminal kinases; MMP-9, matrix metalloproteinase-9; TLR4, Toll-like receptor 4.

Tenascins (TNs) are representative of MCPs and are comprised of a family of four homologs, that is, TNC, TNR, TNW, and TNX (22–24). Among the TNs, only TNC has been investigated in stroke (20). TNC was discovered in the early 1980s and initially referred to by different terms such as myotendinous antigen, glioma mesenchymal ECM, hexabrachion, TN, J1-200/220, cytotactin, and neuronectin (25, 26). TNC is a pleiotropic ECM glycoprotein with a large molecular weight (180–400 kDa). Its N-terminal contains highly conserved heptad repeats, followed by 14 epidermal growth factor (EGF)-like repeats and up to 15 fibronectin type III (FN III) repeats comprised of universal repeats and alternatively spliced repeats; and a fibrinogen repeat domain is located at the C-terminal (25, 27–30). Alternatively spliced repeats in the FN III domain are comprised of a combination of A1, A2, A3, A4, B, AD2, AD1, C, and D domains in humans and A1, A2, A4, B, C, and/or D domains in mice, which are inserted between domains 5 and 6 in universal FN III repeats (20, 23, 29–34). TNC generally forms a disulfide-linked hexamer mediated by the N-terminal domain in which six flexible arms emanate from a central globular particle (**Figure 1**) (20, 22, 29, 30, 35). In humans, TNC is encoded at a single gene located at 9q33 (20).

TNC exhibits a diverse range of isoforms in various tissues, the splicing of which is regulated by intracellular pH. Under exposure to basic pH ~7.30–7.50 as observed with fetal cells and aggressive tumors, the level of longer or larger TNC isoforms is enhanced (33). Isoforms with a large molecular mass (≥200 kDa) contain at least one alternatively spliced FN III repeat. Each alternatively spliced FN III repeat has unique functions (33). Larger TNC isoforms induce cell proliferation and migration, and control cell spreading, resulting in promotion of destruction or remodeling of local tissues (33). In addition, larger TNC isoforms can be easily degraded by matrix metalloproteinases (MMPs), leukocyte elastase, and possibly other serine proteases (33). MMPs usually cleave the sites located within the alternatively spliced region (23, 33). In contrast, TNC isoforms with a lower molecular mass (<200 kDa) lack A1-D domains in alternatively spliced FN III repeats and seem to be more stable in dense connective tissues and to be expressed at low levels in a physiologically normal tissue (33). Under physiological pH <7.0, the level of small TNC isoforms is increased (33). Different TNC isoforms seem to be produced by proteolytic processing of a large multimodular TNC isoform. The proteolytic destruction may impart novel functions to TNC by destroying existing binding sites or generating smaller fragments with new binding sites: these new functions can drive entirely novel processes compared to the previous or intact form (33). Smaller TNC fragments exert quite different reactions in various cells. Fragmented EGF-like domains of TNC induce apoptotic effects on vascular smooth muscle cells in culture, while intact or full-length TNC does not have the functions (36). Thus, respective TNC isoforms seem to have flexible physiological or pathological functions (20). However, the timing and location of distinct TNC isoforms production during inflammatory reactions have not been completely investigated. In addition, the functions of individual TNC isoforms have not yet been fully clarified (20).

### TNC Expression During Developmental Stage

**Figure 2:**
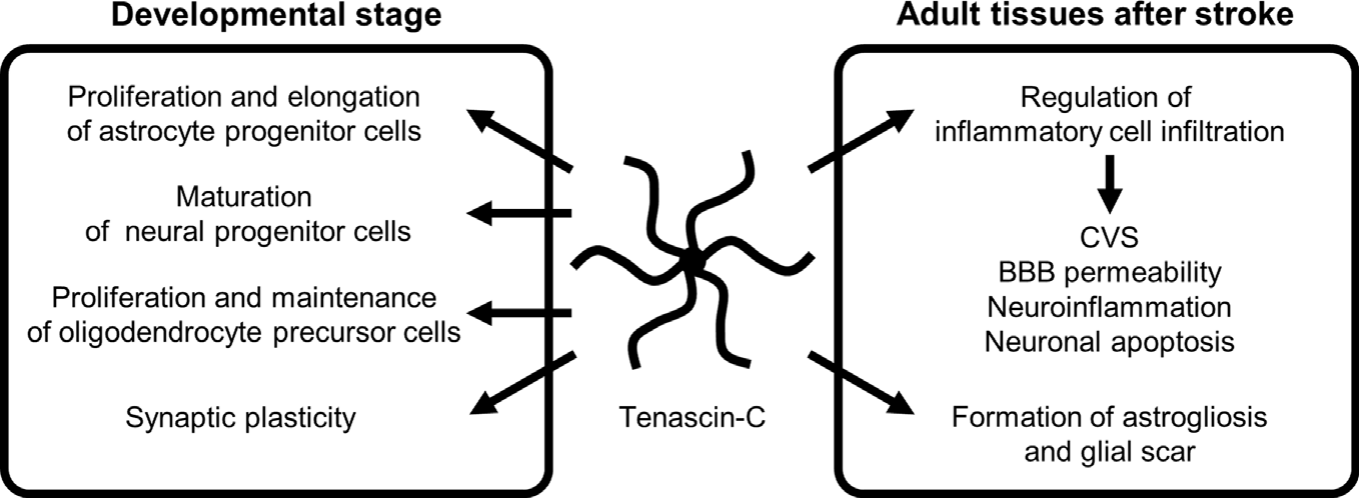
The role of tenascin-C during developmental stage and following stroke. BBB, blood-brain barrier; CVS, cerebral vasospasm.

TNC is highly expressed during embryonic development and was first identified in developing astrocytes (37–41). Currently, TNC is considered to be primarily induced by astrocytes and radial glial progenitor cells and to play a crucial role in normal brain development: it serves as a repulsive substrate for neuronal and astrocytic growth and plays a role in proliferation and process elongation of astrocyte progenitor cells, maturation of neural progenitor cells, proliferation and maintenance of oligodendrocyte precursors, and synaptic plasticity through autocrine and paracrine regulatory mechanisms during developing stages (**Figure 2**) (28, 34, 40–47). In the spinal cord, TNC is synthesized by a subset of gliogenic precursors in the late phase of embryogenesis and influences proliferation and migration of a subpopulation of astrocytes (48). Although the expression of TNC is downregulated in the brain 2–3 weeks after birth, it is involved in hippocampal synaptic plasticity and synchronized neural network activities in the mature brain *via* control of postsynaptic L-type Ca^2+^ channels (47). Intrahippocampal injections of recombinant TNC fragments containing the FN III repeats 6–8 block the retention of memory and hippocampal formation in mice, showing the mediation in hippocampus-dependent contextual memory and hippocampal synaptic plasticity (49).

### Regulation of TNC Expression in Adult Tissues

In adult tissues, the expression and the distribution of TNC are typically limited under normal physiological conditions but transiently upregulated in reaction to inflammatory responses or tissue damages (50, 51). TNC expression is controlled by several transcription factors and intracellular regulators, including T cell factor/lymphoid enhancer-binding factor, nuclear factor (NF)-κB, Notch1 and Notch2, hepatocyte NF-4α, Ets, SP1, c-myc, homeobox transcription factor Prx1, Rho, c-Jun, and extracellular signal-regulated kinases (ERKs) (29, 32). Overexpression of the transcription factors Slug and Sox9 induce TNC and periostin expression (52). However, the involvement of these transcription factors in stroke has not been investigated *in vivo*. In contrast, micro-ribonucleic acids (RNAs) such as miR-355 downregulate TNC expression in breast cancer metastases (53). Upregulation of TNC appears in reactive astrocytes, injured neurons, and glial scar formation with restricted occurrence in space and time: therefore, these cells are considered to release TNC (20, 22, 29, 47, 50, 54, 55). TNC modulates a variety of cell functions and morphologies (22, 23). Scratch wound assays induce TNC expression by astrocytes *in vitro* (56). Levels of TNC enhancement following stab-wound injury to cerebellar and cerebral cortical structures depend on the number of glial fibrillary acidic protein (GFAP)-positive cells, which represent reactive astrocytes (55). GFAP was significantly suppressed in TNC-knockout mice compared to wild-type ones one week after stab injury (57); and therefore TNC may be involved in the late acute phase formation of astrogliosis around sites of injury and failed regeneration (55). However, in another mice study, TNC exerted protective effects after brain damage (57). The study demonstrated that extravasated immunoglobulin G was considerably prolonged and RNA levels of proinflammatory cytokines tumor necrosis factor (TNF)-α, interleukins (ILs)-1β and -6 were higher in the cerebral cortex after stab-wound injury in TNC-deficient mice: TNC production might promote BBB repair or maintain the BBB integrity by the reduction of inflammatory cytokine levels (57).

**Figure 3:**
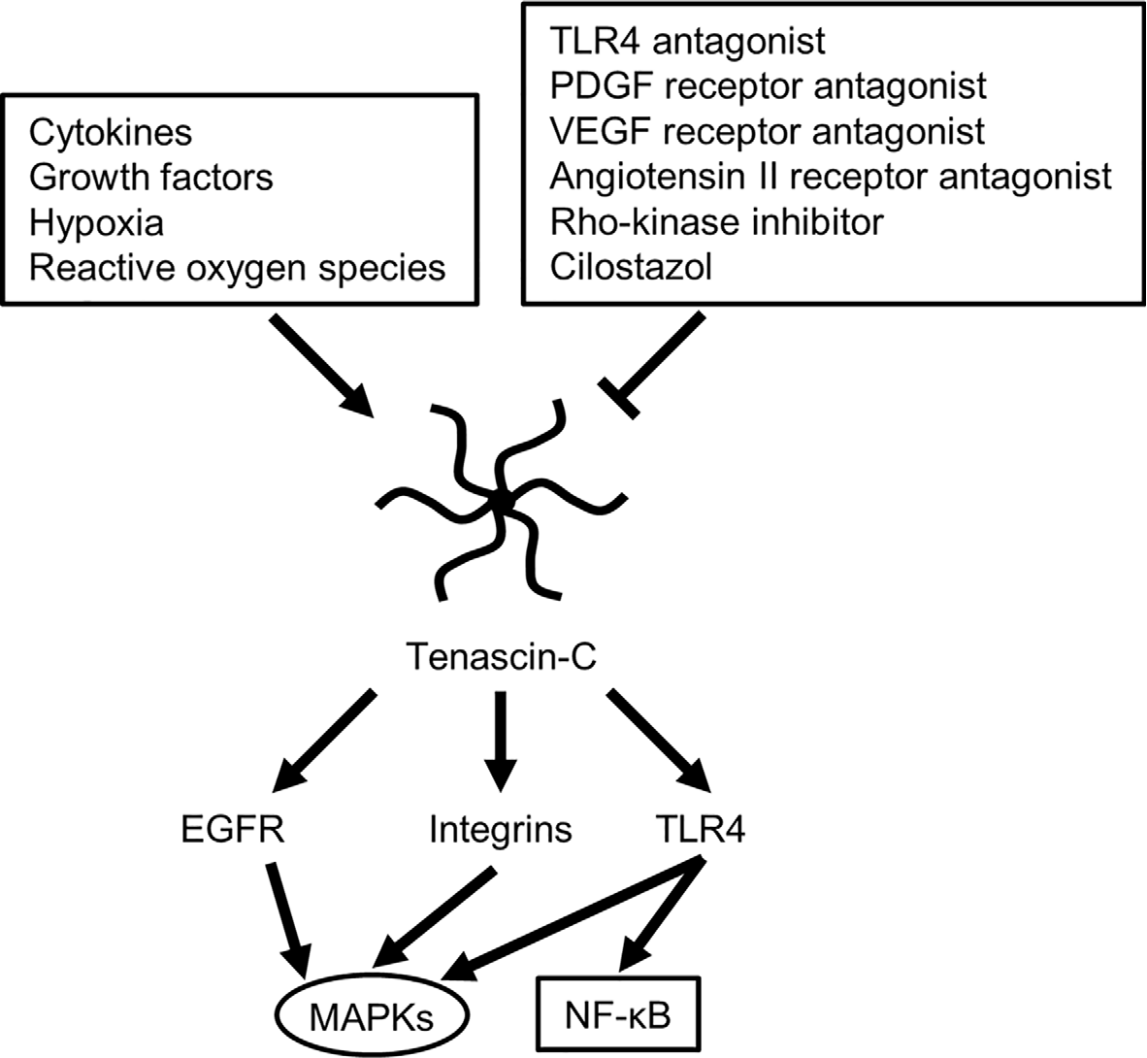
Possible molecular mechanisms for regulating tenascin-C expression and downstream signaling cascade. EGFR, epidermal growth factor receptor; MAPKs, mitogen-activated protein kinases; NF-κB, nuclear factor-kappa B; PDGF, platelet-derived growth factor; TLR4, Toll-like receptor 4; VEGF, vascular endothelial growth factor.

TNC induces MMPs, which seem to result in a positive TNC feedback loop *via* MMP-induced TNC cleavage (29). In addition, many stimuli, including various pro- and anti-inflammatory cytokines, GFs, hypoxia, reactive oxygen species, and mechanical stress, readily but transiently upregulate TNC within several hours in various pathological conditions such as myocarditis, arteriosclerosis, and cancer, irrespective of the location or type of causative insults (**Figure 3**) (29, 58). Clinically, TNC has been reported as a plasma biomarker of neurodegenerative diseases, as significantly elevated TNC levels were found in the peripheral blood of patients with Alzheimer’s disease with mild cognitive impairments and in the amniotic fluid of pregnancies affected by Down syndrome (59–61). In addition, TNC expression is induced in the hippocampi of both epileptic rats and human patients with temporal lobe epilepsy (62–64). In the brains of patients with temporal lobe epilepsy, the regions exhibiting diffuse and elevated expression of TNC were characterized by an extended area of reactive gliosis and synaptic reorganization (42). Loss of TNC in transgenic CRND8 mice caused enhanced production of anti-inflammatory cytokines and decreased production of proinflammatory cytokines, associated with reduction of β- and γ-secretase activity, Aβ oligomerization, amyloid plaque load, and synaptic impairments (65). However, another study demonstrated that TNC may be involved in the maintenance of late acute phase astrogliosis surrounding the site of severe injuries, and exert anti-inflammatory and BBB-repairing effects (57). Thus, TNC induced by reactive astrocytes may play neuroprotective, neurotoxic or other diverse roles depending on the context, including regulation of astrocyte reactivity, BBB permeability, and potentiation of inflammatory processes (**Figure 2**). TNC may also directly affect neuronal plasticity and lead to memory impairments (42).

### TNC in SAH

Many experimental studies as to TNC have been reported in SAH in rats and mice. Some have demonstrated that TNC is expressed in the walls of spastic cerebral arteries (endothelial, smooth muscle, adventitial, and periarterial inflammatory cells) and in brain parenchyma (astrocytes, neurons, and brain capillary endothelial cells), primarily in the surface of the cerebral cortex between 24 and 72 h after SAH by endovascular perforation (22, 37, 66–69). In a clinical setting, TNC levels in the cerebrospinal fluid (CSF) were below the diagnostic threshold level in patients with an unruptured cerebral aneurysm but markedly increased after cerebral aneurysmal rupture (70). Elevation of TNC expression may be affected by several factors, including elevated intracranial pressure as well as brain damage resulting from local or systemic inflammatory reactions (68, 71). A previous experimental study in rats showed that even cisternal saline injections caused elevated intracranial pressure and induced slight subarachnoid inflammatory reactions, which caused TNC upregulation in the basilar artery adventitia (68, 71). TNC is a key pathological factor that promotes activation of inflammatory cell infiltration in the periarterial space, causing EBI in terms of neuroinflammation, BBB disruption, and neuronal apoptosis; and also is involved in CVS and plays an important role in the development of DCI (**Figure 2**) (3–5, 20, 29, 37, 66, 72). Recent studies demonstrated that intracisternal injections of both intact or full-length TNC and recombinant TNC fragments containing the EGF-like repeats which activate EGF receptors activated mitogen-activated protein kinases (MAPKs) in arterial smooth muscle cells, causing prolonged CVS, but had no effects on neurobehavior, brain water content, and BBB integrity in normal healthy rats; however, in SAH rats, the TNC injections caused neurological impairments (3, 16, 22, 66, 72–77). In addition, TNC-induced activation of MAPKs is considered to upregulate MMP-9 in brain capillary endothelial cells and to cause BBB disruption in mice with SAH, although the mechanisms remain unidentified (29, 72, 73, 75, 77, 78). MAPK activation also results in a release of inflammatory mediators (18, 79). Human studies have repeatedly shown elevation of inflammatory mediators such as endothelin-1, TNF-α, and ILs-1β and -6 in CSF after SAH (37, 80–82). IL-1β induces TNC production *via* MAPK-dependent or -independent pathways, while TNC stimulates the synthesis of IL-1β (58, 77). This positive feedback mechanism upregulates TNC and the receptors in an early phase of SAH and may cause more activation of TNC signaling transduction and consequently further development or aggravation of EBI including neuronal apoptosis and BBB disruption, as well as prolonged CVS (**Figure 1**) (66, 69, 73). Post-SAH neuronal apoptosis develops through TNC-induced activation of p38 and ERK1/2 (20, 66), and the EGF-like repeats of TNC have been involved in apoptotic processes in cultured human smooth muscle cells (23). The blockage of TNC induction prevented post-SAH MAPK activation in the brain and suppressed EBI in terms of neuronal apoptosis and BBB disruption (22). Overexpression of ILs-1β and -6 itself is also known to cause apoptosis by triggering caspase cascade reactions (16).

### Effects of TNC Knockout on Experimental SAH

Some studies using TNC-knockout mice have reported a relationship between TNC and EBI or CVS. In a filament perforation SAH model, TNC knockout did not change the total volume of SAH (22). However, TNC knockout alleviated neurological impairment and decreased brain water content and Evans blue dye extravasation, which were associated with inactivation of three major MAPKs (c-Jun N-terminal kinase [JNK], p38, and ERK1/2) in brain capillary endothelial cells in the cerebral cortex; and the MAPK inactivation resulted in inhibition of MMP-9 induction and retention of tight junction proteins such as zonula occludens (ZO)-1 (20, 22, 72, 83). In addition, TNC-knockout mice demonstrated prevention of CVS, which was associated with a reduction in periarterial inflammatory cells infiltration and MAPK inactivation in cerebral arterial smooth muscle cells as well as suppression of caspase-dependent neuronal apoptosis in the cerebral cortex with reduction or inactivation of TLR4, NF-κB, and ILs-1β and -6 (37, 50). TNC knockout also inhibited post-SAH upregulation of another MCP, periostin, in brain capillary endothelial cells and neurons (83). In a hepatic ischemia and reperfusion model, the protective effects of TNC knockout have been also shown in terms of a marked decrease in apoptotic hepatic cells *via* reduction of inflammatory cytokines and MMP-9 (77). Exogenous TNC treatment induced TLR4 and MMP-9 and aggravated EBI in wild-type SAH rats; and abolished the protective effects through induction of TLR4 and MMP-9 in TNC-knockout SAH and transient hepatic ischemic models in mice (20, 22, 66, 73, 76).

### TNC in Cerebral Aneurysm, Post-SAH Chronic Hydrocephalus, and Ischemic Stroke

TNC induced potent aneurysm repair through the fibrosis-promoting effects in a rat aneurysm model, possibly by recruiting macrophages, which secrete cytokines to induce migration and proliferation of smooth muscle cells (84). In contrast, the fibrosis-promoting effects of TNC may cause chronic hydrocephalus after SAH due to obstruction of circulation and reabsorption of CSF (70). Therefore, TNC induction may be protective if it is induced in the ruptured cerebral aneurysm wall but detrimental if it is induced in the brain, cerebral arteries, subarachnoid space, or CSF after SAH (22). However, no studies have investigated the role of TNC in cerebral aneurysmal genesis, growth, or rupture and the subsequent hemostasis. In addition, the effects of TNC on a ruptured cerebral aneurysm itself are unknown. Further studies are needed to clarify the role of TNC in intracranial aneurysm animal models (22).

In ischemic stroke in rats, treatment with neurotrophic factor L-serine upregulated TNC at 5 days post-ischemia and exerted neuroprotective effects by inducing the proliferation of neural stem cells and microvessels and the reconstruction of neurovascular units, resulting in neurorepair in the ischemic boundary zone (85). However, the mechanisms have not been investigated.

### TLR4 Cascades and TNC in Stroke

TLRs are constituents of the innate immune system that are activated by DAMPs. At present, a total of 11 human and 13 murine TLRs have been identified (86). Since its discovery in 1998, TLR4 has been the most studied TLR family member (16, 87). TLR4 signaling is currently considered an important neuroinflammation therapeutic target because TLR4 has the unique ability to trigger two distinct signaling pathways (16, 71, 79, 86–88), the myeloid differentiation primary response protein 88 (MyD88)-dependent cascade in the acute phase and the Toll receptor-associated activator of interferon (TRIF)-dependent cascade in the late phase (86). TLR4 is expressed on the cell surface of various cells including microglia, neurons, astrocytes, brain capillary endothelial cells, endothelial and smooth muscle cells of the cerebral arteries, as well as peripheral blood cells including leukocytes, macrophages, and platelets (16, 86). TLR4 is activated by numerous DAMPs such as red blood cell breakdown products (heme, hemin, and methemoglobin), extravasated fibrinogen and fibrin, various intracellular components, and MCPs including TNC and galectin-3 (**Figure 3**) (16, 18). Activation of TLR4 induces the activation of the adaptor molecule MyD88 and subsequently the downstream signaling transcriptional factors NF-κB and activator protein (AP)-1. The process of AP-1 activation is primarily mediated by MAPKs including JNK, p38, and ERK1/2 (16, 79, 86, 89, 90). Both NF-κB and AP-1 upregulate MCPs including TNC, as well as proinflammatory cytokines or mediators such as TNF-α, IL-1β, -6, -8, and -12, intercellular adhesion molecule-1, monocyte chemoattractant protein, and MMP-9 (16, 20). These proinflammatory cytokines and mediators upregulate specific cell adhesion molecules on endothelial cells and induce neuroinflammation as well as the degradation of the inter-endothelial tight junctions and basal membrane in brain capillaries, which leads to BBB disruption and apoptosis of various cells, aggravating tissue damage after stroke (16, 20, 91). MMP-9 is a proinflammatory mediator induced by inflammatory cytokines and reactive oxygen species, and degrades components of the ECM of the cerebral microvessel basal lamina such as collagen IV, laminin, and fibronectin, as well as inter-endothelial tight junction proteins such as ZO-1, causing BBB disruption (92, 93). TNC amplifies the expression levels through positive feedback mechanisms utilizing the TLR4 signaling pathway, leading to further activation of the signaling transduction and the development or aggravation of secondary brain injury, as TNC itself is a ligand of TLR4 (16, 22). Experimental SAH studies have demonstrated that TNC induces CVS *via* activation of TLR4 and the downstream signaling MAPKs JNKs and p38 for more than 72 h in a rat cerebral artery, and that selective TLR4 antagonists LPS-RS and IAXO-102 inhibit TNC-induced CVS as well as expression of TLR4 in endothelial cells and smooth muscle cells of the arteries (**Figure 3**) (16, 17, 22, 73, 75, 76, 89). Therefore, targeting TLR4 is a potential therapeutic option against neuroinflammation after stroke. A recent study demonstrated that a selective TLR4 antagonist attenuated neurobehavioral impairments and prevented BBB disruption *via* suppression of the expression of MAPK JNK, MMP-9, MCPs such as TNC and periostin, as well as inflammatory mediators such as IL-6 and cyclooxygenase-1 in post-SAH mice (79, 89). TNC-knockout post-SAH mice showed less subarachnoid space infiltration of inflammatory cells in association with suppression of TLR4/NF-κB/IL-1β/IL-6 and the MMP-9 signaling pathway (37, 50, 67).

On the other hand, the late phase TRIF-dependent pathway in stroke induces interferon regulatory factor-3 as well as NF-κB and MAPKs, releasing interferon-β (86, 94). Interferon-β also modulates the innate immune response but exerts both anti-inflammatory and anti-apoptotic effects (94). The ligands of TLR4 interact with the receptor without distinction and induce the same downstream signaling pathways. However, the mechanisms to control the activation of respective pathways remains unclear (86).

## TNC as a Clinical Biomarker of Stroke

In clinical settings of SAH, EBI is very difficult to be diagnosed precisely. Loss of consciousness at ictus, poor initial clinical grade, a large amount of SAH and/or intraventricular hematoma, presence of global cerebral edema, and inflammatory mediators have been generally used as surrogate markers of EBI (20). However, these markers are neither objective nor specific to EBI (35). Highly specific biomarkers that reflect EBI and predict the development of DCI are needed to enable earlier diagnosis and treatment of EBI and DCI (67). The ideal biomarkers should be easily measured *via* simple methods and provide accurate and prompt results (95).

If TNC upregulation after stroke reflects secondary brain injury, blood and CSF TNC concentrations can be a candidate for biomarkers: both concentrations are easily measured using an enzyme-linked immunosorbent assay (35). Previous studies have shown that the level of TNC containing alternatively spliced B or C domains in both CSF and peripheral blood may be used as a diagnostic and prognostic biomarker of inflammation and tissue remodeling processes in several diseases such as cardiomyopathy, myocarditis, osteoarthritis, hepatitis, and tumor (58, 96–100).

In patients with SAH, higher plasma and CSF TNC levels may be associated with severe EBI, angiographic CVS, and DCI (22, 101). Plasma TNC level increases independent of serum levels of C-reactive protein and some proinflammatory cytokines (102). Clinically, the peripheral blood level of TNC isoforms containing a C domain in the alternatively spliced FN III repeats at 1 to 3 days from SAH onset could not predict the development of CVS (68). However, the plasma level peaked between 4 and 6 days from SAH onset and was significantly higher in patients who subsequently developed CVS (68). The plasma TNC level increased before 2.4 days of the development of CVS as determined by transcranial Doppler ultrasonography and before 3.6 days of the onset of symptomatic CVS (67, 68, 103). In intracerebral hemorrhage patients, a higher serum level of TNC containing a C domain in the FN III repeats at admission was associated with greater hematoma volume and worse initial neurological status. In addition, the elevation of TNC level was independently correlated with early neurological deterioration, hematoma growth, and worse clinical outcomes defined as modified Rankin scale score >2 at 90 days (104).

In contrast, the CSF level of TNC containing a C domain in the FN III repeats peaked within the first 3 days after SAH onset and correlated with worse neurological status and greater hematoma volume at admission; and additionally, it predicted the development of CVS and shunt-dependent chronic hydrocephalus as well as poorer functional outcomes (22, 35, 70, 101, 105, 106). The differences in the time course of TNC levels between the plasma and CSF may be because TNC in the CSF may be belatedly transferred to the plasma due to its large molecular weight, although the possibility that TNC is released by different cells between the CSF and the plasma cannot be excluded. Although the reason of different time course of peripheral blood and CSF TNC levels after SAH remains unexplained, the findings in previous studies suggest that severe hemorrhagic stroke may induce higher expressions of TNC and that both CSF and peripheral blood TNC levels could be used in predicting or diagnosing the development of CVS and DCI after SAH (20, 22, 35, 103, 107). At present, the most practical clinical application of TNC appears to be its use as a biomarker (67).

## Contribution of Other MCPs to Regulating TNC Expression in SAH-Associated Neuroinflammation

### Periostin

TNC directly binds to other MCPs periostin and galectin-3, and may regulate the expression levels of each other in stroke, playing diverse roles (29, 42, 54, 66, 101). Periostin is a multimodular N-glycoprotein (93 kDa) with a N-terminal cysteine-rich EMI domain, fourfold repeated fasciclin (FAS) 1 domains in the middle, and a hydrophilic C-terminal region (108). The C-terminal region interacts with other ECM proteins such as TNC, collagen, fibronectin, and heparin (15, 66, 72, 74, 107, 109–111). The FAS1 domain of periostin also directly binds to integrins (αvβ1, αvβ3, αvβ5, and α6β4) and TNC, exerting various functions (112–115). Periostin is secreted by stromal cells, which are stimulated by cytokines, transforming growth factor (TGF)-β, and other GFs which are produced in epithelial cells and other cells (112). In an experimental study, periostin was expressed in brain capillary endothelial cells and neurons in the cerebral cortex at 24 h after SAH induction (83). TNC and periostin may induce expression of each other, forming a positive feedback loop (67, 72, 83, 107, 108). MAPKs are both downstream and upstream of periostin, TNC, and IL-6; and thus activated MAPKs induce periostin, TNC, and IL-6, which in turn activate MAPKs, resulting in a positive feedback to cause and aggravate brain injury *via* various mechanisms including MMP-9 activation (54, 66, 72, 83, 107, 112). An experimental study using an endovascular perforation SAH model in mice reported that upregulated periostin enhanced the expression of TNC associated with activation of MAPKs p38 and ERK1/2 as well as MMP-9, resulting in ZO-1 degradation in brain capillary endothelial cells and the subsequent aggravation of BBB disruption (83). In addition, recombinant full-length periostin administration exacerbated post-SAH neurobehavioral impairments, brain edema, BBB disruption, and TNC induction in the post-SAH brain (83). In contrast, anti-periostin antibody prevented post-SAH neurobehavioral impairments, brain edema formation, and BBB disruption *via* downregulation of TNC, inactivation of p38, ERK1/2, and MMP-9, and the resultant retention of ZO-1 (83, 107). These findings suggest that full-length periostin strongly interacts with TNC and contributes to post-SAH BBB disruption and neurobehavioral impairments *via* the MAPK pathway, and that neutralizing full-length periostin may be an effective novel therapeutic strategy for EBI after SAH (107). TNC-knockout mice also showed the inhibition of periostin induction in the post-SAH brain and exhibited less neurobehavioral impairments (83). The interaction between periostin and TNC may play an important role in post-SAH EBI and provides a new insight for future researches (83).

Periostin also binds to integrins, leading to neuroinflammation and BBB disruption (108). In experimental SAH, the process is at least partly mediated by MAPK activation and upregulation of MMP-9 (108). However, periostin-integrin binding also induces neurogenesis *via* activation of the phosphoinositide 3-kinase (PI3K)/Akt signaling pathway and upregulation of an anti-inflammatory cytokine TGF-β (18). The apparent discrepancy may be resolved by future studies to clarify how periostin relates with each integrin subtype in cerebrovascular diseases. In a clinical setting, a higher serum periostin level at admission was associated with worse initial neurological status, greater hemorrhage volume, more frequent development of DCI, and worse clinical outcomes in patients with aneurysmal SAH (116). In addition, plasma periostin levels increased before the development of DCI, irrespective of the presence or absence of CVS (108, 117). Therefore, periostin levels in the peripheral blood may be a predictive marker for post-SAH DCI, regardless of CVS development.

### Galectin-3

Galectins are a family of MCPs comprised of more than 15 members of the β-galactoside-binding lectins and their conserved peptide sequence elements in the carbohydrate-recognition domains (CRDs) which show high affinities to β-galactoside-containing carbohydrate moieties of glycoconjugates (118). Galectins are classified into three types: proto-type (galectins-1, 2, 5, 7, 10, 11, 13–20), tandem-repeat-type (galectins-4, 6, 8, 9, 12), and chimera-type (galectin-3). Proto-type is comprised of monomers or homodimers with the sole CRD; tandem-repeat-type consists of N- and C- terminal distinct CRDs connected by a single-polypeptide-chain linker; and chimera-type has a C-terminal CRD and a N-terminal non-CRD domain which consists of proline- and glysine-rich short tandem repeats (118). The characteristics of chimera-type galectin-3 is to form a bridge between different ligands and to provide different functions (118). Recently, some studies exhibited that galectin-3 is activated through binding to TNC *via* its CRD domain (20, 118, 119). Activated galectin-3 possibly causes the development of brain injury including neuroinflammation after stroke (118–120). Galectin-3 induced by pro-inflammatory mediators contributed to brain immune responses *via* a major inflammatory signaling of Janus kinase/signal transducer and activation of transcription (STAT) and NF-κB pathways (121–123). In addition, galectin-3 is a ligand of TLR4 and activates its downstream signaling pathways as described above (18). In clinical settings, higher acute-stage plasma galectin-3 levels were associated with the development of DCI with no angiographic CVS after SAH (120). An experimental study showed that galectin-3 might cause post-SAH BBB disruption possibly by binding to TLR4 and activating ERK1/2, STAT-3, and MMP-9 (124).

### Osteopontin

Osteopontin, another MCP, seems to have inhibitory effects against TNC in the setting of SAH (19). Osteopontin is an acidic phosphoglycoprotein (40–80 kDa) that contains several functional domains, allowing for integrin and CD44 receptor binding (15, 35). Osteopontin is subjected to numerous post-translational modifications including serine/threonine phosphorylation, glycosylation, tyrosine sulfation, and transglutamination, all of which regulate its functions (15). Five distinct isoforms are generated by alternative splicing (15). Thrombin and MMPs-2, -3, -7, -9, and -12 induce proteolytic cleavage of osteopontin (15). Osteopontin regulates homeostasis, angiogenesis, and immune responses through the upregulation in a variety of diverse cell types at the site of injury, stress, and inflammation (125). An intracellular form of osteopontin was expressed in dendritic cells and macrophages of the immune system in response to transient ischemic injury in the brain, and a secreted form of osteopontin promoted remodeling of the ECMs in the brain (15). After SAH induction, osteopontin binds to L-arginyl-glycyl-L-asparate (RGD)-dependent integrins and exerts neuroprotective effects by alleviating CVS and BBB disruption *via* induction of MAPK phosphatase-1, an endogenous MAPK inhibitor (22). Interestingly, both osteopontin and RGD-dependent integrin receptor antagonists significantly inhibited the vasoconstrictive effect by recombinant TNC fragments containing EGF-like repeats (20). The findings suggest that RGD-dependent integrins may be involved in CVS development, and that TNC binds to the integrins to develop CVS. Although the mechanisms of osteopontin’s anti-TNC effects remain poorly understood in stroke, osteopontin and TNC share some receptors such as RGD-dependent integrins, and therefore at least partly competitive inhibition may be the mechanism (22). A novel multimodal nanoparticle, simultaneous multiple aptamers and RGD targeting, which combines triple affinity for nucleolin, RGD-containing integrins, and TNC, has been reported as a candidate for a targeted therapy against TNC (126): the nanoparticle would be well worth trying in SAH and other stroke types, considering the possible effects on both RGD-dependent integrins and TNC.

## Contribution of GFs and Integrins to Regulating TNC Expression in Stroke

The FN III domains 1–5, specifically domain 5 of TNC, have a high binding affinity for multiple GFs, such as platelet-derived GF (PDGF), vascular endothelial GF (VEGF), fibroblast GF (FGF) including FGF-2, and TGF-β1 as well as neurotrophin-3 (**Figure 1**) (27, 30, 54).

### PDGF

PDGF is a homodimeric, non-glycosylated, polypeptide chain GF with a molecular weight of 28-35 kDa (127, 128). In SAH studies, PDGF is upstream of endogenous TNC and interrelated with TNC (66, 67, 69, 74). Exogenous TNC injections induce and activate PDGF receptors (PDGFRs) possibly *via* interreceptor interactions, which in turn upregulate TNC in the cerebral arteries and brain (66, 69). TNC may be further upregulated by a positive feedback on more PDGF activation *via* upregulated PDGFRs and crosstalk signaling between receptors, leading to more MAPK activation and consequent development of CVS, neuronal apoptosis, and neurological impairments in SAH rats (22). In rat SAH models, an intraperitoneal injection of imatinib mesylate, a tyrosine kinase inhibitor of PDGFR, showed the suppression of TNC induction and attenuated neurological impairments, the development of CVS and neuronal apoptosis *via* inactivation of MAPKs such as JNK, p38, and ERK1/2 (22, 66, 69). In addition, a cisternal injection of recombinant TNC to imatinib mesylate-treated experimental SAH rats reactivated MAPKs to abolish the protective effects of imatinib mesylate on neuronal apoptosis and CVS, resulting in neurological aggravation (22, 66, 69). Thus, TNC downregulation was demonstrated to be involved in the neuroprotective effect mechanism of imatinib mesylate (72), and PDGFs and PDGFRs were suggested as a potential therapeutic target to regulate TNC expression and to prevent post-SAH EBI and CVS (**Figure 3**) (67).

### VEGF

VEGF, a member of a family of secreted polypeptides with a highly conserved receptor-binding cystine-knot structure similar to that of the PDGF, is a homodimeric protein (34–46 kDa) that stimulates the formation of blood vessels (129). Although TNC regulates VEGF expression in tumors, no studies have reported if VEGF directly induces TNC (74, 130). In mice, VEGF enhances BBB permeability in normal brain as well as brain with inflammatory diseases (74). Neutralization of VEGF downregulated VEGF receptor-2, a major mediator of the kinase activity effects of VEGF, in association with suppression of TNC expression and MAPKs activation (69, 73, 131). Taken together, TNC may be involved in VEGF-induced BBB disruption in SAH (**Figure 3**) (72).

### FGF-2

FGF-2 belongs to the FGF family and exhibits several isoforms with molecular weights ranging 18–34 kDa (132–135). FGF-2 is highly expressed in the brain and regulates a variety of cell functions including proliferation, morphogenesis, and suppression of apoptosis (27, 136, 137). FGF-2 is secreted by damaged neurons, and the synergistic action with TGF-β1, which is also upregulated in response to an injury, stimulates the expression of TNC (138). TNC binds to FGF-2 and promotes survival of oligodendrocyte precursor cells by enhancing FGF receptor-mediated signaling and blocking bone morphogenic protein signaling (139). A recent study showed that recombinant FGF-2 activated PI3K and Akt, leading to suppression of neuronal apoptosis after SAH (132). Thus, administration or augmentation of FGF-2 may be a promising therapy to reduce post-SAH neuronal apoptosis *via* activation of the FGF receptor/PI3K/Akt signaling pathway (132). However, the action of FGF-2 on TNC after stroke has not been investigated.

### Integrins

Integrins are a superfamily of cell adhesion receptors that primarily recognize ECMs and cell-surface ligands, and are composed of α and β subunits that form 24 known combinations (140). Five members of the integrin family that recognize TNC as a ligand have been identified: isoforms α2β1, α8β1, α9β1, αvβ3, and αvβ6 (140, 141). All the integrins except for α9β1 bind to the FN III repeat sites of TNC, while α9β1 binds to the fibrinogen globe (20, 140). Integrin αvβ3 is expressed on endothelial cells and activates the downstream signaling that involves MAPKs, proinflammatory mediators such as ILs, and MMP-9; however, the role of the integrin αvβ3 signaling pathway in stroke has not been investigated (19, 37, 83, 106, 140, 142–145). Activated integrin αvβ3 induces internalization of ZO-1 and occludin, disrupts vascular endothelial-cadherin localization, and increases expression of MMP-9 (146). Therefore, activation of integrin αvβ3 may be involved in BBB disruption. In contrast, β1 integrins form laminin-binding, collagen-binding (α2β1), RGD-binding (α8β1), or ECMs-binding (α9β1) heterodimers (147–150). The β1 integrins are increased in cerebral blood vessels in ischemic cortex, and induce angiogenesis as well as leukocyte adhesion and migration following ischemic stroke (147, 151). In addition, increased β1 integrins in neuronal cells were associated with neuronal adhesion, and neurite outreach and regeneration (151). Thus, it has been demonstrated that β1 integrin signaling is required for neurovascular formation and recovery as well as endothelial cell migration, proliferation and blood vessel formation following transient ischemic stroke in mice (152). Therefore, β1 integrin may be a therapeutic target for ischemic stroke and other pathological conditions through modulating angiogenesis (152). On the other hand, α2 integrins have been reported to be associated with an increased risk for ischemic stroke (151). Activation of α2β1 integrin prevents endothelial cells from proliferating through binding to laminin (153). In addition, overexpression of integrin α2β1 was associated with ischemic stroke and myocardial infarction by clot formation, while its absence results in a prolonged bleeding time within safe limits (154). Therefore, inhibition of integrin α2β1 may be a potential therapy for ischemic stroke. The expression and the role of integrins α8β1, α9β1, and αvβ6 have not yet been elucidated following stroke (147). At present, it is unknown if integrins influence TNC expression.

## Other Therapeutic Candidates for TNC-Induced Brain Injury Following Stroke

TNC expression can be reduced by several medications, including cilostazol, steroids, and non-steroidal anti-inflammatory drugs (NSAIDs) (**Figure 3**) (32, 103, 155). An *in vitro* study found that cilostazol, an anti-platelet and peripheral arterial vasodilating agent, is a selective inhibitor of phosphodiesterase type III with pleiotropic actions that include the inhibition of inflammatory reactions (18, 155). Blockage of phosphodiesterase type III can inhibit induction of TNC at the transcriptional level by activating the cyclic adenosine monophosphate–protein kinase A signaling pathway (103, 155). In patients with aneurysmal SAH, 300 mg/day cilostazol treatment almost completely suppressed the elevation of plasma levels of TNC variants containing alternatively spliced FN III B and C domains at days 1–12 after SAH onset, and prevented the development of DCI and chronic shunt-dependent hydrocephalus, resulting in improved clinical outcomes (103, 156). TNC is induced by inflammation, and TNC itself can induce inflammatory reactions (58, 66, 157). Therefore, some anti-inflammatory medications are also associated with reduced TNC expression. For example, steroids and NSAIDs suppressed TNC expression in macrophages and human vascular smooth muscle cells *in vitro* and in arterial smooth muscle cells *in vivo* (32). With respect to inflammatory signaling, in a rat model of SAH, a MAPK JNK inhibitor SP600125 reversed the vasoconstrictive effects of TNC, and a MAPK p38 inhibitor SB203580 abolished TNC-induced TLR4 upregulation and TNC’s vasoconstrictive effects (73).

Angiotensin II is a well-known potent inducer of TNC but the potential mechanisms have not been identified (158). Drugs that inhibit the effects of angiotensin II such as angiotensin II receptor blockers (ARBs) may block vascular TNC expression (159). In a model of carotid artery stent implantation in hypercholesterolemic rabbits, an ARB candesartan cilexetil prevented in-stent neointimal hyperplasia, which was associated with a decrease in macrophage infiltration and TNC expression in the arterial wall: the immunostaining study showed that TNC was induced in a limited area around the stent struts, but the expression disappeared by the ARB treatment (109). ARBs may suppress in-stent restenosis after carotid artery stenting *via* anti-inflammatory effects through TNC inhibition (109). Eplenerone, an aldosterone receptor antagonist, also inhibited the development of inflammation and fibrosis associated with reduced TNC expression in an angiotensin II-induced hypertension model in mice (160).

Inhibition of Rho-kinase also suppressed expression of TNC in smooth muscle cells in hypertensive rat pulmonary arteries (161). In a clinical setting, a Rho-kinase inhibitor hydroxyfasudil is commonly used to prevent CVS after SAH in Japan, although the levels of TNC have not been measured (20).

Currently, Neuradiab^®^ (81C6 anti-TNC antibody; Bradmer Pharmaceuticals, Inc.) and double-stranded RNA directed against TNC have been reported as candidates for anti-TNC directed therapy (126). Further evidence would facilitate the development of therapeutic agents targeting TNC.

## Conclusions

TNC potentially plays a key role in pathophysiological changes *via* neuroinflammation and appears to be a future therapeutic target in patients with stroke. However, the protective and detrimental roles of TNC with respect to each disease and the stage have not been completely unveiled. If TNC is set as a therapeutic molecular target, the therapeutic (time) window should also be addressed. Current evidence shows that TNC can be a biomarker to predict secondary injuries following stroke. Further studies to determine the underlying molecular mechanisms of TNC-induced pathophysiological changes and the regulation of TNC expression are warranted.

## Author Contributions

Both authors contributed equally to the planning, preparation, drafting, and writing of the article. All authors contributed to the article and approved the submitted version.

## Funding

This work was funded by the Taiju Life Social Welfare Foundation (Grant Number, N/A) and the JSPS KAKENHI (Grant Number JP20K09346) to HS.

## Conflict of Interest

The authors declare that the research was conducted in the absence of any commercial or financial relationships that could be construed as a potential conflict of interest.
